# Addressing Racism and Its Deeply Entrenched Dynamics: A 21st Century Imperative

**DOI:** 10.1089/heq.2022.29019.rtd

**Published:** 2023-01-13

**Authors:** Gail C. Christopher, Algernon Austin, Mindy Thompson Fullilove, Alan Jenkins, Charmaine D.M. Royal, Lisa Sockabasin

**Affiliations:** ^1^ Executive Director, National Collaborative for Health Equity, Washington, District of Columbia, USA.; ^2^ Director, Race and Economic Justice, Washington, District of Columbia, USA.; ^3^ The New School, New York, New York, USA.; ^4^ Professor of Practice, Harvard Law School, Cambridge, Massachusetts, USA.; ^5^ Robert O. Keohane Professor of African & African American Studies, Biology, Global Health, and Family Medicine & Community Health, Duke University, Durham, North Carolina, USA.; ^6^ Wabanaki Public Health and Wellness, Bangor, Maine, USA.

***Dr. Christopher:* Hello, everyone. I am Gail Christopher, executive director of the National Collaborative for Health Equity (NCHE), and I am just so humbled and honored that you as our senior scholars have written these wonderful articles for us and are going to engage now in a roundtable discussion of these critical issues. I want to begin by saying that the issue of racism, the grounding I call it meme and ethos of our culture has been danced around for too long by far too many people and our politics, our governance infrastructure, our investments, and certainly, the opportunity structures to be healthy are all shaped by this organizing theme of our society, which is hierarchy of human value, or racism.**

**I am really excited to be engaging in this very frank discussion with each of you who are such scholars and who can help to shed light in a way that, as I say, calls people into the conversation more so than calls people out because we need as many people as possible in this discussion and willing to make a stand for us to transform our country. I am honored that the Robert Wood Johnson Foundation has funded our leadership work, and that the W.K. Kellogg Foundation while I was there had the courage to launch a National Truth Racial Healing and Transformation Initiative.**

**We, unlike many other countries in the world, have failed to really face the truth of our past and to embed a commitment to facing that, redressing it, and moving forward. So, the truth, racial healing, and transformation are the organizing comprehensive framework for our work at the National Collaborative, and many places around the country are doing this work, and it has five pillars.**

**Each of you has written a beautiful article that explores one of those pillars. The first one is narrative. And it is certainly the issue of the stories that we tell about our country, about ourselves, and the significance of that, particularly in driving our perceptions and our scientific middle view, as it were. We were so pleased that Dr. Charmaine Royal took on this narrative change conversation for this particular effort.**

**I am going to invite Charmaine Royal to tell us why narrative change for health is so critical to achieving health equity.**

**Dr. Royal:** Thank you, Gail. It is such a pleasure to be part of this conversation today; I appreciate the invitation to engage with this group. I am a professor of African and African American studies and biology at Duke University, also working in global health and family medicine. I am trained in human genetics and bioethics, and bring those disciplines together in my research, scholarship, and teaching. My work focuses on the ethical and social implications of human genetics research, particularly issues at the intersection of race and genetics. The narrative change pillar is directly related to my work. As Gail alluded to earlier, the stories we tell and who tells those stories, shape our historical and current realities. The narratives that I focus on in my research and in my brief have to do with what race is, what it is not, and how it is related to racism and adverse outcomes.

For centuries, the dominant narrative has been that humans are naturally divided into distinct biological races, and that there is a hierarchy among these races, with people of primarily European ancestry being superior to everyone else. My work and the brief that I prepared are focused on helping to change that narrative, helping people understand that we made up this story. Science has long confirmed that regardless of our diverse physical characteristics, we as humans are one species—*Homo sapiens*. Unlike some organisms, there are no biological divisions within our species that can be defined as subspecies or races.

Although biological human races do not exist, racialized human groups do, and it is important to understand the differences between these concepts. The invention of race, racial classifications, and racial hierarchies in humans were driven by a need to justify economic, political, and social domination of groups deemed to be inferior based primarily on physical features. Racism created race and is the root cause of the associated inequities and disparities we see today. This narrative must replace inaccurate narratives about the relationship between race and racism and the reasons for differences in health and other outcomes among racialized groups. Now, I am not naive enough to think that replacing prevailing false narratives alone is going to fix the racism problem. But it is critical to the process, because beliefs influence attitudes and attitudes influence behaviors.

We have done research that has demonstrated that more than 50% of the U.S. population believes that there are biological human races. Some people also believe that there is a racial hierarchy. Furthermore, research has shown that biological race thinking influences how we do scientific research, how we provide health care, and how we live in society. To achieve health equity, we must dismantle beliefs in the existence of biological human races and racial hierarchies that underlie and perpetuate structural inequalities and health disparities. The need to promote more accurate and complete narratives about race and racism has never been greater or more urgent. And science must be part of the conversation in my view. It will take science and society working together to change narratives, minds, and hearts and lead us to transformation.

***Dr. Christopher:* Thank you so much for that. And we are going to come back to some of the critical issues that you raised, but one of the key takeaways for me was that racism created race and that this is a joint effort, science, and all aspects of society that must commit to eliminating that fallacy. So, thank you so much for that. And the next topic we are going to discuss is racial healing. And from a practical standpoint, would you please answer the question, Lisa Sockabasin, in terms of why is racial healing such an important precursor or a necessary component to achieving equity and fairness and more optimal health outcomes?**

**Ms. Sockabasin:** Kci woliwon, Gail, for the question. My name is Lisa Sockabasin. I am a Passamaquoddy citizen from Wabanaki territory, which is what we now call the state of Maine. My work has always been centered on Indigenous people, our culture is our prevention, our culture is our medicine, and providing services and developing programs that really work in our communities. I am the co-CEO of WPHW. It is an organization here in Wabanaki territory that is growing quite fast. When I joined just a little over 4 years ago, we were less than a dozen people trying to make profound change in our community.

Four years later, we are close to 200 people, 70% Indigenous, wildly enthusiastic about healing in our community. When we think about racial healing, when we think about the relationships that are needed for that, we look at the massive amount of work before us. We see a nation that is utterly divided, not just divided, aggressively divided. And we also see this deep disconnection with our land that we share, and we do not own. Our land is now poisoning us because we have poisoned her. Our relationships are deeply disconnected.

What I believe is that when we heard Charmaine talk about the stories our history has told in this country, one of those stories that has been missing for far too long is that Indigenous story. Those stories in the books that describe us as uncivilized and savages when the reality is we should be measuring civilization based on how much love is present rather than what we are accomplishing based on capitalism. The racial healing that needs to happen, these relationships that need to be restored, we need so much now more than ever the people that we made invisible in this country, Indigenous people. That perspective of love first always is what is informing how we build health care for our people, how we build education and recovery programs, knowing that wellness and healing is easy when love is present and impossible with the hatred that we now are all experiencing.

So, my article is very much focused on the Indigenous perspective of relationships, the knowing that the concept of race is silly. We are all connected. We all have roles, and they are different, just like the scholarly roles today, but equally as important. So, I hope what you see from my article is this charge, this calling in that you describe Gail, this calling in for censuring all of us, each other even when there is difference, finding what are those things that we can work toward, and believing that what this country tried so hard to make invisible is the very strength that can pull it together today.

***Dr. Christopher:* Thank you so much, Lisa. And our diversity, our Indigenous values, it is our strength as a nation and as a world, I would even say, as a global family. Because each and every one of us will trace our roots back to an Indigenous people, and the decision to deny that is a lethal decision truthfully. So, I thank you so much for those comments, and we will come back to explore that in more depth as well.**

**Our next scholar, Dr. Mindy Fullilove, is going to explore how the tool of separating and dividing has been embedded in our systems and structures, the five pillars of the THRT framework are narrative change, racial healing and relationship building, separation, the law, and the economy.**

**Dr. Fullilove:** Good morning, everybody. Nice to be with you. I am Dr. Mindy Fullilove. I am a social psychiatrist on the faculty at the New School. As of this day, we are currently on strike fighting for decent pay for our part-time faculty. The New School is behaving atrociously, so we are all trying to support the picket line. And it is one of those things that we are talking about: how you can mistreat people by giving them a special name so they are not real faculty, they are part-time faculty. With demeaning name, you can give them terrible pay and no benefits and make them perform piles of unpaid work. One of the things that I learned when we were working on a project to observe the anniversary of Jamestown in 2019—our 400 years of Inequality Project—was that just at the time that Africans were being brought over and sold into slavery, the colonists were stealing the land from all the tribes that lived in that Jamestown region.

People described this as, “Stolen hands working stolen lands,” that these processes of theft were happening at the same time. At that same time, they were also bringing women over to be indentured servants but really to be sex slaves. All of these forms of inequality emerge at the same time and are interwoven. Anybody can be labeled something and then situated in this hierarchy, which is then applied to the land. Lisa said we do not own the land, in fact, land owns us. But the commodification of the land in 1619 transforms the land from the source of life, the mother of us all, into something you buy or sell. The commodification of people and the commodification of the land are happening at the same time.

Which leads to the first point I would like to make about separation: the importance of redlining, a policy instituted in the 1930s, but very evident to this day in the traces it has left and the impact it had on the American city. Redlining is said to be about undermining black neighborhoods, but really it authorizes the disinvestment in the whole American city. This kind of illusion is the way in which racism is used to hide the real objective, which is to make as much profit as you possibly can and to use people as you use the land without concern for long-term sustainability.

The second point is that there is a paradox in separation. The more you divide people, the more tightly they become connected. This is an unexpected finding that is presented in the important ecological work of doctors Roderick Wallace and Deborah Wallace. We think we are separate, but in fact, we are one, and we are all suffering from the ways in which this stratification is imposed upon us and destroys our relationships, destroys our strengths, and destroys our health.

And then the third is that racism is constantly evolving, because oppressed people are constantly fighting it. Women have fought it. Jews have fought it. Native people have fought it. African Americans have fought it. Everybody fights it. It is disassembled, but then it tries to invent new ways to assert itself, and we are certainly in this period of the new racism lifting its ugly head and tearing at us. You pick up the paper every day, and there is another incident of racial hatred, most recently in Colorado Springs. So, it means that none of us are safe at any time. This new racism, as Lisa was describing, is so angry and so aggressive and has been promoted by the top levels of the U.S. government. Part of the question we face now is, how are we going to respond to something that is all pervasive, connects all of us while we feel disconnected—so that is a terrible paradox—and it is constantly evolving?

One of the things I wanted to throw into the conversation was a concept that Doctors Wallace who had done all this ecological thinking on apartheid said, look, you cannot address a problem this big with any simple solution. You have to have a solution, what they called the magic strategy, which operates at multiple levels of scale and in multiple systems. I really appreciate their proposal about magic strategies. They are trying to teach us that we have to think ecologically because we are in an ecological crisis. As Einstein said, “You can't solve the problem with the thinking that created it.” We can't solve the problem of racism with the thinking that created it, that sees people as separate. We have to see us all as in one kettle, one pot, one something.

***Dr. Christopher:* Beautifully said, Mindy. Your article is profound in that expression and communication, and I am going to invite our next scholar, Alan Jenkins, to talk about the law, which is a broad, broad topic, and we know that in this pillar, as are all of them. But again, why is addressing this structured legal dynamics so important if we are going to achieve our ultimate goal of people having the opportunity to be healthy and to live up to their potentials?**

**Prof. Jenkins:** Thanks very much, Gail. I am Alan Jenkins. I am a professor of practice at Harvard Law School. In my previous lives, I have been a civil rights attorney, a human rights grant maker, a social justice leader at a communications lab, and now here as a scholar of the law. The law is one of the principal ways in which we organize ourselves as societies. We establish what our societal values are, how we treat each other, and how our government will interact with us and vice versa. And in the United States, the law has been both an implement for oppression and extreme discrimination and also at times, at the best of times, an instrument for greater and more equal opportunity, human rights and human dignity.

In my article, I briefly traced some of that history. That our founding documents declared the remarkable proposition of equality and freedom and a government by, for, and of the people. And at the same time, it embedded the principle of human chattel slavery tied to race. It both assumed and furthered the attempted genocide of Indigenous peoples. It intentionally excluded women and many other people in our nation. And that was the case for the first century almost of our existence as a nation. I should note that the Supreme Court, to whom we will return, was most frequently an instrument in furtherance of discrimination and oppression, declaring, as Lisa noted, Indigenous people to be “savages,” unworthy of either controlling or even remaining on the land that they had inhabited since time immemorial, declaring that black people, whether enslaved or free, had no rights that whites were bound by the law to respect.

And so it was for many, many decades, the fundamental change coming with the Civil War and importantly the Reconstruction amendments to our Constitution—the 13th, 14th, and 15th amendments to our Constitution—that many of us, both historians and those of us who study the law through legal academia, see as a fundamental transformation of the relationship between people, the states, and the federal government.

With those amendments, we saw an attempt at a transformative multicultural democracy. At the end of a bloody Civil War and centuries of slavery, we saw the Reconstruction Congress, the radical Republican Reconstruction Congress, grant and protect the right to vote for, at that time, black men; declare that equal protection under the law is the law of the land; and abolish not only slavery but also its incidences and things relating to it. We saw it briefly for about 12 years, a transformation of African American men elected to Congress and to state legislatures, of real attempts—often through the use of federal troops—to ensure the right to vote and other aspects of equality.

Then, the nation lost interest, and troops were withdrawn with the Compromise of 1877. Jim Crow discrimination was installed. Throughout that period, the displacement, the disrespect, the persecution of Native and Indigenous peoples continued. The period of around the turn of the century between the 1800s and the 1900s was considered the nadir of human rights in our country, with that transformative vision resurrected in the 1950s and 1960s and into the 1970s with civil rights laws passed.

Activism was always driven by the people, driven by people of color, driven by women of all races, and later in the century, gay and lesbian, LGBTQ+ Americans. And now we are in a period in which the nation has again lost interest in that progress, and it is really up to all of us to reignite that spark.

In my article, I talk about some things in the short term, in the medium term, and in the long term that all of us can do to promote greater equity and inclusion and respect for human rights—which are, of course, deeply tied to health and health equity for everyone.

Many of the short-term items relate to things that this current Biden administration is doing in terms of a very important executive order that requires the spending of trillions of dollars of federal funds in a way that advances equity, of the Interior Department under the leadership of Deb Haaland, the first Native American Cabinet Secretary in the modern era, to include tribal voices and input and guidance in Interior Department decision-making, to examine our shameful legacy of boarding schools and the trauma that has caused, to advance and lift up tribal government solutions to addressing climate change—both from a resilience standpoint and from a prevention standpoint. These are all short term, meaning that they live and may expire with this administration, but present remarkable opportunities for all of us to engage and move the ball.

The article touches briefly on the rise of reparative laws. Evanston, Illinois, most remarkably, has passed what we believe is the nation's first reparations ordinance tied to housing opportunity, in particular, and derived from taxes on the cannabis industry.

And then a call finally for long-term transformative change, finishing the business of the original Reconstruction through what many of us call a “Third Reconstruction,” the second one being in the 1950s and 1960s, which is going to be a long-term enterprise, but is really going to be about that realignment of the people's narrative, as Peggy Cooper Davis and colleagues have called it, rather than a Confederate narrative of states' rights. Rooted in anti-subordination, rooted in full and equal opportunity, equality, and human rights. And I fully believe that we can get there as a nation given our social movements and leaders of the day.

***Dr. Christopher:* Thank you, Alan. And thank you so much for having hope and belief that we can get there. I think that is a very important segue to hear from our final scholar, Dr. Algernon Austin. And we know that the engine for this culture that we all live in, the engine is an economic one, and so that is the final pillar of this framework, this comprehensive approach to eliminating racism and its consequences.**

**Dr. Austin:** I am Algernon Austin, the director for Race and Economic Justice at the Center for Economic and Policy Research. Although we talk about the pillars separately, every time I think about them, and it is reflected in my work, they are all connected. As Dr. Fullilove talked about, there are no small policies that are going to address this. We really need all the pillars working simultaneously. They are all intertwined with each other. So right now, in terms of thinking about the economy and its impact on health equity, I want to really drill down a little bit on one of the points in my article.

The article was structured on certain principles in terms of understanding racial inequality and racial equity. One of the points that I put forth was that economic inequality contributes to racial inequality. I want to talk about that now more explicitly in terms of health equity. Our public health researchers know and have informed us that the social determinants of health can be more important than actual health care in determining the health outcomes of a population. Among the social determinants that these public health researchers examine include income and poverty, unemployment and job security, working conditions, and other economic factors. The public health researchers are telling us that economic factors are critically important to understanding the health outcomes of a population. I am not a public health researcher, but I can imagine the mechanisms working in ways such as the following.

For example, many Americans are constantly stressed because they do not know how they are going to pay their bills. About half of all renters are in what we call unaffordable housing because they are paying more than 30% of their income on housing. About a quarter of these renters are paying more than 50% of their income on housing. If you are paying more than 50% of your income on housing, you are likely struggling to stay afloat. You are worrying about being evicted, worrying about being homeless, juggling bills from day to day. There is a lot of day-to-day stress in your life. And we know that chronic stress leads to negative health outcomes. People with chronic stress are more likely to gain weight. They have high blood pressure. They suffer from heart disease. They have immune system problems. They suffer from insomnia, experience anxiety and depression, and have other health problems.

So, America's failure to have an effective affordable rental housing policy is likely subjecting anywhere from a quarter to a half of the rental population to chronic stress and increasing this population's likelihood of having negative health outcomes. This failure in affordable rental housing policy has inequitable outcomes from a racial perspective. So here, the class inequality that is reflected in our housing policy then contributes to the racial inequality in health outcomes. For example, although only a minority of white households are renters, a majority of black households are renters. So, this failure of affordable rental housing policy has a disproportionate negative impact on black households. The stress and negative health consequences of not being able to afford rental housing falls disproportionately on black households. So, here we can see the racial inequity effects of this economic policy failure.

***Dr. Christopher:* Thank you so much. Thank you to each of you. And in this next question, I am going to ask you to sort of step back. And if you had a moment—and the power—to bring about a change in our society that would disrupt this pattern—and Alan, you spoke so vividly about how that one period in history would seem to be the nadir for the efforts toward equity and fairness and justice. And Mindy, you described how it has to be a holistic essentially approach. There is no simple thing. And we were reminded by Lisa of the Indigenous power, the culture is the medicine, so to speak. And of course, Charmaine reminded us of the embedded nature of this fallacy of a belief in a hierarchy of human value that it is embedded in the science, which we pride ourselves, as Western minds. We pride ourselves that the science is objective, and it drives.**

**And of course, Algernon, your illustration of the housing is absolutely relevant, particularly in this post-COVID era. And thank you for reminding us that although we have a framework that has pillars, it is all interconnected. But for each of you, could you think about what do you think is the prescription? What do you think we could do that would put in place the infrastructure for disrupting this episodic dynamic that we have had? What could we put in place that would make this a sustainable, continuous, focused, passionate effort? Knowing our two-party system, knowing the level of polarization that we have right now, if you could call for something that would be transformational, but most importantly, would not be subject to the ebbs and flows of political will, is there a thing? What do you think that could be? And I invite all of you to respond to that one.**

**Dr. Austin:** So, in my thinking, we have to get the American people of all races to get beyond a zero sum framework. People are constantly assuming that if something has a benefit for another group, it is going to harm their group. And that really prevents us from creating policies that lift up and benefit the common good. This zero sum framework is deep within our culture. Unfortunately, there are people who are invested in fostering it. But if we can somehow get people to stop seeing the other group as the enemy, as us versus them, then we can work on solving our problems collectively. But it is a real challenge. But the benefit is also real. And we must communicate this. This must be part of the narrative change.

For example, just recently this year, the consulting firm McKinsey and Company estimated that closing the black/white wealth gap could grow the economy by 1 to $1.5 trillion. So that $1.5 trillion would benefit people of all races. But when we enact the policies to improve the outcomes of African Americans, unfortunately, we know that many people will say, well, if that is benefiting African Americans, it is taking something away from me. And I am going to oppose it, not being able to see that closing this gap will actually lead a 1 to $1.5 trillion benefit for people of all races. It is a lot of work to get people to see the big picture, the long-term picture and not be stuck in this us versus them zero sum framework.

***Dr. Christopher:* Thank you very much, Algernon. Anybody else want to weigh in on that?**

**Dr. Royal:** Algernon said pretty much what I was going to say. It is the mind and heart work, getting people to truly embrace our common humanity, interconnectedness, and interdependence, realizing that what benefits you benefits me and what hurts you hurts me. And separation is one of the most powerful tools of racism. The more we separate people, keeping them from interacting with others who we have racialized and who we think are so different, the less likely it is that we are going to get to know, understand, and become empathetic toward one another. We are one human family, with a beautiful tapestry of diverse physical features and cultural values and experiences. The variation in our physical characteristics is part of our humanness. It is important for people to understand that at so many levels, we need each other.

***Dr. Christopher:* I appreciate both of your perspectives. Anybody else want to weigh in on that question in terms of what is it going to take?**

**Dr. Fullilove:** I would like to weigh in. I do not know that there is anything that is a permanent change. Racism is like COVID, right?

***Dr. Christopher:* It adapts, right.**

**Dr. Fullilove:** COVID is getting out from under the vaccines and our natural immunity. We are in a cloud of COVID variants. The pandemic is over, but we have COVID. Racism is like that. We get into the same kind of crazy binds because we are so ambivalent—not all of us, but the powers that be are so ambivalent about letting go of any of the power that they get from keeping us divided. But you know, Abraham Lincoln said, “You can fool all the people some of the time, and you can fool some of the people all the time, but you can't fool all the people all the time.” Racism is about fooling people, and you cannot fool all the people all the time.

One of the things that I thought about a lot in writing my book *Urban Alchemy* was about alchemy, magic. Some of what happens in human social conditions is a little bit beyond linear. There are other dimensions to it, which we could call magical. Look at what happened to the elections. The Republicans were going to sweep until they overturned *Roe*, and then they did not. They never thought that was coming, right, as the next thing. Human beings are interesting and complicated, and we have all these group forces and all these things in conflict. New threats make us think in new ways. We are facing the sixth extinction, right? But it gives people some new ideas. I think we need to keep showing up every day and keep challenging the lies. We need to promote love, and we have to reconnect people to the Earth.

**Ms. Sockabasin:** When I was hearing Algernon speak, what was in my head was this: we have been colonized to competition, this scarcity mentality that we hold that is a poison. And I described our organizational growth earlier. We grew really fast, and we grew really fast because we would not compete, and we had an abundance mindset always. We knew, as Charmaine said, we need each other. That means even the people who disagree with us. That means even the people who might not like us. Now, sure, healthy boundaries is always important to keep yourself safe. However, this intolerance of difference has really put us in a difficult place. I think about what does it look like in action.

Well, you can create huge movement when you take an abundance mindset, when you are calling people in, as Gail said, and doing less of the call out. That does not mean we do not hold people accountable. It simply means we can hold them with love, even if they have done something that has been hurtful. And I just believe that is the only way we are going to get through this. I think about this little town in Maine that we created this Center for Wabanaki Healing and Recovery, this multisite campus in a rural town in Maine that is very sacred to Indigenous people. It is the home of Mount Katahdin, a sacred mountain that we pray to, and we have prayed to for thousands of years.

We hold ceremony here still to this day. So that is where we wanted to go back to heal, to recover. And it has been difficult to be welcomed into this small town. Our promise was to the town from the beginning regardless of what may have been thrown at us was that we were going to love this land and the people on it, no matter what. And so, when there was a racial incident in the town where people were hurt based on a public display of mockery for a celebration, Juneteenth, that means so much to us, there was vitriol thrown at this business that put up the sign, as you would expect. There was hatred, there was destruction, there was violence, threats to this family that, yes, put up the sign. And although I understood the anger, I did not understand the response. It is so hard to see, I believe, anyone destroyed like that publicly or privately. So, what our organization did is we opened up our doors.

**Ms. Sockabasin:** We said what we need in this community is healing. And we want everyone inside, even the family that put up the sign. And I met with them before—I spent over 2 hours. There were a lot of tears. There was a lot of accountability. There was a lot of learning. And there was also love. They came out in public to a healing circle held by us with the townspeople. They are the very people that they said they could not go to the grocery store anymore. They could not walk to the post office. Both of those incidences left trauma with them because it was difficult for people for what was done and for the people who did it. So how do we heal?

There are just such deep wounds that we are not going to heal with how we are showing healing publicly. So, I do not know what structures need to be in place, Gail. But what I do know is that accountability looks different than we are seeing.

***Dr. Christopher:* Powerful, powerful. Thank you so much. Alan, from a historic and a legal structural standpoint, does anything come to mind?**

**Prof. Jenkins:** I agree with Mindy that probably the only thing permanent is change. So, I do not know if there is something to for all time and forever establish full and equal opportunity and rights. But I do know, at the same time, I think, number one, we have learned a lot about the enterprise that I think almost all of you have mentioned of narrative change and actually moving hearts, minds, and policy in a lasting way. Many of us have been on that journey, either apart or together, of really learning what people respond to, learning what our common stories are, learning what shared values can help us move forward, values that many of you have mentioned: community, the idea that we are all in it together, abundance, opportunity for sure.

And that leading with those shared values instead of dry facts or vitriol or anger is crucial to bringing people in while moving forward in the right direction, that telling a systemic story that is, of course, core to a public health approach, but it is often not the way our issues get discussed. And, therefore, many audiences go immediately to a personal responsibility narrative that blames people for the obstacles that they face or the outcomes that they experience, rather than understanding that it is all of our responsibility to create structures that respect rights and dignity and allow opportunity and mobility and equity for everyone. And leading with solutions.

We often used to say at The Opportunity Agenda, my old shop, that there is a reason why Martin Luther King Jr.'s greatest speech was not called “I Have a Complaint.” But we often forget about that, especially those of us who are advocates. And people in the United States and around the world are in no mood to hear about more problems to which there is no answer. It is really incumbent upon us to talk in terms of values, problem, solution, and action. And that is critical. There are a number of organizations, The Opportunity Agenda, John Powell at the Haas Institute, the Narrative Initiative, and others, which are working to better tell our story.

And the last thing I would say is more rooted in the law. We also know that there are avenues for structural lasting change. The one I will point to is our international human rights system, which is tattered but survives, and which recognizes that full racial equality is critical to the fulfillment and experience of all rights and for all humanity. And yet our court systems, our government has—after helping to create that system in the 1940s, have turned away from so much of it, from virtually all of the economic, social, and cultural rights; from the notion that discrimination in effect and in impact is as important as intentional discrimination; from the sovereignty of Indigenous peoples.

I think that is one goal for us to work toward as we try to move hearts, minds, and policy. Embracing that body of international human rights laws and principles is one of those—we call it the top of the spigot strategies. If you can turn that on, then rights and equality will flow to many other places where they currently do not exist. Those are all difficult to do, but I think we are better equipped to do them than probably at any other time in our history.

***Dr. Christopher:* Wow. You all have given eloquent and powerful answers to that question. And it was a tough question. I really want to thank you from the multiple perspectives that you shared, all integrated, all interrelated, which is what we know is at the heart of this work. It is like the human body. We have specialists who work on the brain or work on the heart. But we know it is all interrelated, and we need a holistic approach to solve any problem. I want to just ask you to acknowledge if you would in this next question, this is the 21st century. It is a cultural ethos that is in many ways different from prior centuries. I jokingly say there are some advantages to growing older. None of them are physical.**

**But you live through this the 1950s, 1960s, 1970s, 1980s, 1990s, and into the 21st century, and you have seen the changes—when I say you, I mean me. I have seen the changes that have happened. We are in a culture now that is unique. And I would love for each of you to give some thought to the cultural nuances of today that would enable a transformation, if we look back at Dr. King, and we look at the Civil Rights Movement, and we look at the tactics and the campaigns of the 20th century, which became viable because they found their way into our living rooms through television. It was at the peak of television right that we got exposure to what was happening in those campaigns in the South. But we live in a different moment right now where we have social media, and we have algorithms, and we have toxic algorithms, and we have artificial intelligence.**

**And we have this rare moment of an administration that is committed to equity. So, I wondered if each of you could respond to what we need to leverage in this 21st century culture that could drive our movement. You know, there is an African proverb that says, “To stumble is not to fall but to move forward faster.” What could we leverage right now that would move us forward faster in addition to the wonderful answers that you have already given? Is there something about this culture, this moment? Is it climate change? Is it the postpandemic? Is it artificial intelligence? So I would invite any of you to respond to that one knowing that is probably not one thing.**

**Prof. Jenkins:** I think one of the reasons that I am optimistic is because our current culture is a culture of social movements and a culture of intersectional social movements, which has really not been the case, especially in the United States, through much of our history. Just in the past two decades, or decade and a half, we have seen the Occupy Movement, the Black Lives Matter movement, the #MeToo, and #Time'sUp movements. We have seen the transgender rights movement. We have seen movements that have integrated, for example, environment and tribal sovereignty, such as the pipeline opposition. And today's younger folks, at least younger than I am, ever since Kim Crenshaw wrote her seminal article, we have been talking about intersectionality of race and class and gender and gender identity and ethnicity.

The movements led by young people are talking the talk and walking the walk. There is a seamless connection for them between those identities and the challenges that folks are facing. A fascinating example for me was the gun safety movement that was re-energized after the terrible shootings at Marjory Stoneman Douglas High School. Of course, today, this week, we are mourning the deaths of additional people. But those kids who were mostly middle class and relatively privileged came out as leaders and immediately recognized their privilege and made sure that they were including as peers and fellow leaders, black and brown young people from low-income communities who had been dealing with the fallout of gun violence in much larger ways and for much longer.

And those movements joined. The same thing with the #MeToo movement, which was founded by Tarana Burke, but then it was Alyssa Milano, a white, relatively privileged celebrity, who began the social media movement. But immediately, folks tapped her on the shoulder and said, you know what, your voice is important, but it is not the only voice here. And that movement became much more intersectional. I think we need to lean into that hard. And by we here, I mean those of us who are in professional positions, who are researchers, who are teachers, lawyers, and lawmakers. There is a huge amount of inspiration and vision and genius to be had there. I think we need to support those movements. We need to provide guidance when it is asked for and also to listen and learn and be led. What allows me to sleep at night is the vibrance and genius of those social movements, which I think really characterize our current era, almost like none we have seen.

***Dr. Christopher:* Brilliant. Thank you so much. And so it is a culture of movements right now and how that is leveraged.**

**Dr. Austin:** I agree with Alan. I think he had some great points. He was very optimistic, but I would like to give a somewhat pessimistic spin on that and then try to be more optimistic. I think the culture of today, social media in particular is very effective at fracturing us and keeping us divided. We know that the algorithms aggressively segment us and separate us. And it is also the social media space where it is very easy for disinformation and misinformation to circulate, disinformation and misinformation that fuels racial animosity, racial conflict, and racism. So, it presents a real challenge. I may be showing my age in arguing that we need to go back to some of the older ways. But I do not think it is simply my age. There have been analyses that found that the conservative movement actually behaves more like the progressive movement did in the past, and that is part of the conservative movement's effectiveness.

Some of the right wing movements that foster racial animosity are more willing to meet people where they are and work to bring people into their movement. We have talked about the importance of calling people into movements. I think progressives need to do more of this. Some of these right-wing movements start by connecting people on grounds of common interests, and then later persuade them to support right-wing viewpoints. You know, I have a gun, you have a gun, let us go to a gun club, and then later people are converted into accepting extremist right-wing political positions. Anand Giridharadas has written about this issue from a different perspective in his latest book titled *The Persuaders* Today, I think, progressives rely too heavily on social media.

Progressives think that if we like the same tweets or whatever, therefore, we are a movement. And that is all we are going to focus on. Whereas I feel some of the retrograde forces are actively trying to pull people into their movements through interpersonal interactions more. I think we need more of what Lisa had talked about earlier, about really calling people in and reaching out and having that sort of in-person interaction, which can be so powerful at transforming people. Also, It is hard to have wild stereotypes and misinformation about a person when you are interacting with them regularly. That can be very powerful, and we cannot let a lot of social media followers cause us to ignore the power of that interpersonal interaction.

**Ms. Sockabasin:** I cannot agree more than what has been stated about our young people. Our young people are the ones who have grown up benefiting from the movements before them. They are the ones who are impacted by the violence in our schools. They are voting. Those voices are so important. I would say the other piece of this is I would be remiss if I did not mention that our world is on fire. Our Earth is struggling as much as we are. That is what happens.

And not only does our Earth struggle and we struggle, our animals do, too. It is hunting season here in Maine, so you hear the stories of the animals that are just too sick to even harvest after being hunted. And so, what used to sustain us is now sick. We have to be moving with a sense of urgency, and I know there is a movement to do less and slow down. And what I am saying is now is not the time, folks. This is why we need each other so badly, every person who is here, is because I understand that people are too busy, but I am just asking let us just move faster to freedom, which is let us be strategic with our busyness. Because right now, our Earth needs us.

***Dr. Christopher:* Thank you so much, Lisa, for reminding us of that fundamental freedom that we have, that fundamental choice that we have that we will either live or die based on our relationship with our Mother Earth. Charmaine, any thoughts about this moment from your world of genomics and science? Is there something in this cultural moment that we should be seizing?**

**Dr. Royal:** Science is constantly reinventing itself and evolving. And given its bidirectional relationship with society, science has been engaging in its own national and global reckoning with racism in this cultural moment. In genetics, specifically, there has been a notable increase in efforts (some led by our professional societies) to confront and make amends for the role of the field in manufacturing and maintaining race and racial hierarchies. In addition, and related to this, is a growing recognition of the need to incorporate environmental factors into genetics research.

This not only improves the quality of our science, but also helps dispel biological race thinking by conveying the important message that health, disease, and other outcomes are influenced by both genes and environments. I cochair a National Academies committee that is examining the use of race, ethnicity, and ancestry as population descriptors in genetics research. I cannot talk about our recommendations that are getting ready to come out soon. But from that work and other efforts, it has been heartening to hear geneticists say we cannot fully understand genes outside of the context of the environment. So, as science and other disciplines advance, they are recognizing and taking steps to address their limitations and interdependence.

***Dr. Christopher:* Thank you so much for that. That is a wonderful sort of reminder that we are catching up with our ancestors, so to speak, in terms of Western science, is seeing what Indigenous people knew and said all along. I really appreciate that perspective. Mindy, Dr. Fullilove, it seems to me we might have a burgeoning movement for labor and for fairness. I am wondering if there is something in that makes this moment unique and unique opportunity.**

**Dr. Fullilove:** There are quite a lot of labor movements. The railroad workers may go on strike. The whole University of California system is shut down right now. Apartheid is so important to the power structure, they always want to talk about race and they want to suppress any issues of class. We do live in a capitalist society where the capitalists own everything, and the workers are exploited. Every country in the world has shows on TV now. I have been particularly drawn into Korean dramas, which for me as a psychiatrist are fascinating because they are always stories of highly traumatized people and how they get healed and get reintegrated into society. The very ancient culture that has dealt with lots of upheaval and displacement, but also a very brutal transition to capitalism in just 70 years.

There are stories about how do we hold on to the things in our culture that are precious to us while becoming an economic miracle? These are fascinating stories, and it gets to that what do we do about fairness, what do we do about inclusion? It is not all about money. It is not all about goods. It is not all about commodities. At the heart of it, we have to be thinking about people. That is what they stress in those stories. But there are also stories from India, from China, and from everywhere. And so that is just fascinating as the world has opened up and come into my living room.

***Dr. Christopher:* All of your answers are powerful in terms of are describing this unique moment. And I think our opportunity and our challenge is how do we move forward in a clear eyed way, recognizing where we are now, what we have at our fingertips, and how do we leverage and in some ways, for want of a better term, even exploit the opportunities in this moment or seize them, as they say, seize the day to accelerate our pace for transformation and change. If there is one final sentence or words that you want to express about your vision, Alan, what are you hopeful for?**

**Prof. Jenkins:** I think this is a moment in which people are newly open to many many possibilities. And that makes it both a dangerous time but also a time that is full of opportunity for transformative positive change. There are things, from reparations to universal basic income to fundamentally re-envisioning our justice system, which would have been immediately dismissed just a few years ago that are now on the table. They may or may not occur, but there is the opening to dream big. And I think one of the biggest mistakes that we could make, especially those of us who live and work in institutions, is to dream too small, because of the extent to which we have been beaten down by past cynicism about big ideas, to fail to advance those ideas today. And I think if we do not advance our big ideas, someone else will, and we will be living in potentially a more problematic more dangerous world. And I also just want to finally say thank you so much for including me.

***Dr. Christopher:* Any of you want to summarize your hope in a brief statement, given what we have all shared and where we are right now?**

**Dr. Royal:** I think about public education and academic and cocurricular education, helping people better understand race and racism, what they are, and what they are not. There are some excellent initiatives such as the museum exhibit, “Race: Are We So Different?” that was created by the American Anthropological Association in 2007, and traveled across the country for more than 10 years. In 2017, the exhibit was here in North Carolina at the Museum of Natural Sciences, where it drew one of the largest numbers of visitors to the museum. Some people were asking why is a race exhibit at a science museum. But this exhibit includes information about the history of race, the role of science in that history, and the experience of living with race. It is helping people understand the concept of race itself and its impacts on individuals and society, and is changing their thinking.

Given the public interest in the exhibit, the state of North Carolina now owns one of the long-term displays of the exhibit. A colleague of mine, Brian Donovan, studies K-12 biology education, and his research has shown that how we teach about human genetic variation and genetic inheritance influences how children think about race and difference. Specifically, he and his colleagues found that teaching accurate information about group differences and the role of genes in outcomes can reduce the prevalence of racial bias.

I teach an undergraduate course on race and genetics at Duke. Over the years, many of the students have said that course was the first time they were hearing that the human species is not divided into biological races. At the end of the course, when I ask them how they see us making sustained progress with addressing race and racism, the most common response is that the teaching of this information needs to start earlier—in elementary school or before. Education about who we are as humans, how and why we have come to be different, and what those differences mean and do not mean with regard to race is effective in helping students and the broader public better understand the multiple dimensions and consequences of race and racism. More importantly, it enhances our collective capacity to positively transform America and the global community.

***Dr. Christopher:* That is a powerful vision that our curriculum—especially at a time when books are being banned.**

**I appreciate that reminder, that that is really where it happens, where we reach the minds and the hearts and the shape the perceptions and the attitudes. And we can diminish the fears, you know, and we can build up the resilience so that people are not so vulnerable to the lies. Thank you so much, Dr. Royal, Charmaine, and for your participation in this inaugural group of scholars. And I am going to invite anyone else to share their vision for the future, where you would like to see this change happen.**

**Ms. Sockabasin:** When I heard Alan talk earlier, one of the things that really brings me deep hope is seeing more of those people who had been made invisible in our society extremely visible, whether it is the vice president of our country, to the cabinet, including Secretary Deb Haaland. We are seeing visibility in places where I think some never thought they would see them. And that is amazing. And then I think about what Mindy said about the power of television. And I think about Indigenous people are being seen in shows now, and we are telling our story and shows that non-Indigenous people are loving as well. So that Indigenous story, our connections to this land, that visibility in all places gives me hope that this land will continue to be protected.

**Dr. Austin:** There are so many brilliant minds and so many people doing really amazing things. There is an important point in terms of the narrative about the economy that we need, I think, for the transformation. Lisa talked about the harmfulness of the scarcity mentality, and Alan talked about the flip side of that–recognizing abundance. Too often, when we do economic policy, we are led to ignore the fact that we are an incredibly rich country. We have tremendous resources. We can make the transformations that we need, but we are always convinced that there is not enough, that we can't, we can't, we can't. But in fact, we can. We are incredibly rich. We can address climate change. We have the technology. We have the skills. We have the resources. We just need the political will. And, because climate change affects all of us, I am hopeful that it can break us out of harmful us-versus-them thinking.

***Dr. Christopher:* Dr. Mindy Fullilove, you have the last word. What gives you hope?**

**Dr. Fullilove:** Well, the ancestors, that beautiful story that Frederick Douglass was feeling discouraged, and Sojourner Truth said to him, “Frederick, is God dead?” And that was in 1847, so imagine what they had to go through before they ended slavery and all the things that they went through for the rest of their lives. So, if they could do it, we could do it.

***Dr. Christopher:* I am just so thrilled by this roundtable, by the brilliance that you all have brought to this discussion. And just a heartfelt thank you.**

## Expert Panel

**Dr. Gail Christopher** is an award-winning social change agent with expertise in the social determinants of health and well-being and in related public policies. She is known for her pioneering work to infuse holistic health and diversity concepts into public sector programs and policy discourse. Dr. Christopher recently retired from her role as senior advisor and vice president at the W.K. Kellogg Foundation, where she was the driving force behind the America Healing initiative and the Truth, Racial Healing and Transformation effort. Dr. Christopher also served as Kellogg's vice president for Program Strategy and worked on place-based programming in New Orleans and New Mexico. In 1996, she was elected as a fellow of the National Academy of Public Administration. In 2015, she received the Terrance Keenan Award from Grantmakers in Health. She currently chairs the Board of the Trust for America's Health. In 2019, she became a senior scholar with George Mason University's Center for the Advancement of Well-Being. Dr. Gail Christopher also became the executive director of the National Collaborative for Health Equity in 2019. In 2021, Dr. Christopher was elected by the American Public Health Association (APHA) Governing Council to serve as the APHA honorary vice president for the United States.

**Dr. Algernon Austin** is the director for Race and Economic Justice at the Center for Economic and Policy Research. Algernon Austin has conducted research and writing on issues of race and racial inequality for over 20 years. His primary focus has been on the intersection of race and the economy. Austin was the first Director of the Economic Policy Institute's Program on Race, Ethnicity, and the Economy where he focused on the labor market condition of America's workers of color. He has also done work on racial wealth inequality for the Center for Global Policy Solutions and for the Dēmos think tank. At the Thurgood Marshall Institute, the think tank of the National Association for the Advancement of Colored People (NAACP) Legal Defense and Educational Fund, Inc., he worked on issues related to race, the economy, and civil rights. Austin has a PhD in sociology from Northwestern University, and he taught sociology as a faculty member at Wesleyan University. He has discussed racial inequality on PBS, CNN, NPR, and other national television and radio networks. His most recent book is *America Is Not Post-Racial: Xenophobia, Islamophobia, Racism, and the 44th President.*

**Dr. Mindy Thompson Fullilove**, MD, is a social psychiatrist and professor of urban policy and health at The New School. Since 1986, she has conducted research on AIDS and other epidemics of poor communities. With a special interest in the relationship between the collapse of communities and decline in health. She has published over 100 scientific articles and 8 books. Among her books are the highly regarded Urban Restoration Trilogy, *Root Shock: How Tearing Up City Neighborhoods Hurts America and What We Can Do About It*, *Urban Alchemy: Restoring Joy in America's Sorted-Out Cities* and *Main Street: How a City's Heart Connects Us All*.

**Alan Jenkins** is a professor of practice at Harvard Law School, where he teaches courses on Race and the Law, Communication, and Supreme Court Jurisprudence. Before joining the Law School faculty, he was president and cofounder of The Opportunity Agenda, a social justice communication lab. Jenkins's prior positions have included assistant to the solicitor general at the U.S. Department of Justice, where he represented the U.S. government in constitutional and other litigation before the U.S. Supreme Court; Director of Human Rights at the Ford Foundation, where he managed grantmaking in the United States and 11 overseas regions; and associate counsel to the NAACP Legal Defense and Educational Fund, where he defended the rights of low-income communities facing exploitation and discrimination. He previously served as a law clerk to U.S. Supreme Court Justice Harry A. Blackmun and to U.S. District Court Judge Robert L. Carter. Jenkins is a frequent commentator in broadcast, print, and digital media on topics ranging from Supreme Court decision-making to racial equity to the role of popular culture in social change. His past Board service includes New York Public Radio, the Center for Community Change, the Legal Action Center, and Futuro Media Group, as well as the Board of Governors of the New School for Public Engagement. He has also served on the Selection Committee for the Sundance Documentary Fund. Jenkins holds a JD from Harvard Law School, an MA in media studies from the New School for Public Engagement, and a BA in psychology and social relations from Harvard College.

**Dr. Charmaine Royal** is the Robert O. Keohane Professor of African & African American Studies, Biology, Global Health, and Family Medicine & Community Health at Duke University. She directs the Duke Center on Genomics, Race, Identity, Difference and the Duke Center for Truth, Racial Healing & Transformation. Dr. Royal's research, scholarship, and teaching focus on ethical, social, scientific, and clinical implications of human genetics and genomics, particularly issues at the intersection of genetics and “race.” Her specific interests and primary areas of contribution include genetics and genomics in African and African Diaspora populations; sickle cell disease and trait; public and professional perspectives and practices regarding “race,” ethnicity, and ancestry; genetic ancestry inference; and broadly defined genotype–environment interactions. The fundamental aim of her work is to dismantle ideologies and systems of racial hierarchy in research, health care, and society. She serves on numerous national and international advisory boards and committees for government agencies, professional organizations, research initiatives, not-for-profit entities, and corporations. Dr. Royal obtained a bachelor's degree in microbiology, master's degree in genetic counseling, and doctorate in human genetics from Howard University. She completed postgraduate training in ethical, legal, and social implications research and bioethics at the National Human Genome Research Institute of the National Institutes of Health, and in epidemiology and behavioral medicine at Howard University Cancer Center.

**Lisa Sockabasin** is a citizen of the Passamaquoddy Tribe at Motahkomikuk with extensive experience in tribal, state, and federal governments, nonprofits, and philanthropic organizations. In her capacity as the co-CEO of Wabanaki Public Health and Wellness (WPHW), Lisa collaborates with tribal leadership, the WPHW team, and federal and philanthropic partners to address systemic inequities experienced by Wabanaki communities in Maine and to develop and implement culturally based programs that respond to the needs of our communities. Lisa has over 20 years of experience addressing inequities experienced by and providing opportunities to tribal populations. Before joining WPHW, Lisa served as the director of the Office of Health Equity in the Maine Department of Health and Human Services, as an epidemiologist in the Infectious Disease Program for the State of Maine, as a nurse epidemiologist with the North American Indian Center of Boston, Inc., and served for two decades as coordinator to the Brigham and Women's Hospital and Harvard's Four Directions Summer Research Program. Lisa holds a BS in biology from the University of Maine, a BS in nursing from the University of Maine, and an MS in Health Policy and Management from the University of Southern Maine. She holds a graduate certificate in nonprofit management and serves on several boards, including Maine Philanthropy Center, Planned Parenthood of New England, the American Civil Liberties Union (ACLU) of Maine, and the Maine Development Foundation.

